# Large Group Exposure Treatment: a Feasibility Study in Highly Spider Fearful Individuals

**DOI:** 10.3389/fpsyg.2016.01183

**Published:** 2016-08-09

**Authors:** André Wannemueller, David Appelbaum, Maike Küppers, Amelie Matten, Tobias Teismann, Dirk Adolph, Jürgen Margraf

**Affiliations:** Mental Health Research and Treatment Center, Department of Clinical Psychology, Ruhr-Universität BochumBochum, Germany

**Keywords:** exposure therapy, group treatment, participant modeling, one-session treatment, spider fear treatment, spider fear

## Abstract

A large group one-session exposure treatment (LG-OST) based on indirect modeled exposure strategies was carried out to investigate its feasibility and effectiveness in a sample of highly spider fearful individuals (*N* = 78). The stability of LG-OST-effects was assessed at 8-month follow-up (FU). Furthermore, a second sample (*N* = 30) of highly spider fearful individuals was treated in a standard, single-person one-session treatment (SP-OST) design to compare LG-OST-effects to a standard spider fear treatment. Participants’ fear of spider was assessed by multiple questionnaires and by a behavioral approach test. The fear assessment took place before and after the respective intervention, and at 8-month FU in LG-OST. Regarding subjective spider fear measures, LG-OST mainly showed medium to large effect sizes, ranging from Cohen’s *d* = 0.69 to *d* = 1.21, except for one small effect of *d* = 0.25. After LG-OST, participants approached the spider closer at post-treatment measures (*d* = 1.18). LG-OST-effects remained stable during the 8-month FU-interval. However, SP-OST-effects proved superior in most measures. An LG-OST-protocol provided evidence for feasibility and efficiency. The effects of LG-OST were equal to those of indirect modeled exposure strategies, carried out in single-settings. LG-OST may represent a useful tool in future phobia-treatment, especially if it can match the effects of single-setting OST, e.g., by including more direct exposure elements in future large group attempts.

## Introduction

In Europe, patients’ demand on cognitive behavioral psychotherapy is likely to increase in the future (Ginger, 2009). However, the existing health care system has not kept up with the growing demand and cannot sufficiently cover the urgent need for treatment. In some rural areas of Germany, for instance, a psychotherapist is expected to cover the needs of 23.000 inhabitants (Walendzik et al., 2010). Patients are forced to accept long waiting periods and might face avoidable ‘chronifications’ of disorders. Therefore, more efficient treatment approaches to improve clinical effectiveness should be developed.

With a 12-month prevalence rate between seven and nine percent in western countries (American Psychiatric Association, 2013) Specific Phobias are the most common amongst anxiety disorders. Although widely considered as less restrictive, the disability level resulting from different phobic disorders is highly variable ranging from relatively minor (i.e., spider phobia; snake phobia) to more debilitating (i.e., emetophobia; dental phobia). A survey conducted by the German Federal Ministry of Health (Wittchen and Jacobi, 2004) showed the high health-economic relevance of specific phobias by reporting a mean of 4.2 days of absence from work per month for men and 2.6 for women suffering from a Specific Phobia. The population mean of monthly absence was reported to be 0.9 days.

Short and intense exposure-based treatments are already a part of the treatment of many anxiety disorders, especially phobic disorders. In some cases such treatments consist of a single session lasting for 3 h or even less. Originally such one-session treatments (OSTs), first introduced by Öst (1989), consisted of exposure *in vivo* and elements of participant modeling. However, cognitive and motivational aspects have been added through psychoeducative elements, skills training, reinforcement, and cognitive challenges (Zlomke and Davis, 2008). During a typical OST, the therapist encourages the patient to interact with the feared stimulus by mastering a step of a subjective fear hierarchy. The therapist challenges patient’s beliefs in the context of the fear-evoking stimulus and motivates him to emulate. Later on, therapist’s support is restricted to instructions and presence only.

One-session treatments have successfully been applied to a wide range of specific phobias, i.e., animal phobias including spider phobia (Öst et al., 1991; Hellström and Öst, 1995), flight phobia (Öst et al., 1997a), dental phobia (Haukebø et al., 2008), injection phobia (Öst et al., 1992) and agoraphobia (Öst et al., 2001). Across phobic disorders, OSTs show clinical improvement rates of 80–90% (Zlomke and Davis, 2008), proving a very high efficacy in the treatment of adults.

In spite of the short and effective treatment format, the efficiency of OSTs is restricted by the typical single-setting design. Therefore, it would be beneficial to reduce the therapist-patient ratio, i.e., via group treatment, to maximize treatment-efficiency.

Indeed, there are three studies, in which one session treatments were delivered in a group setting. They all were conducted in small groups of spider fearful individuals. Two of these studies (Öst et al., 1997b; Götestam, 2002) focused on concepts about the delivery of exposure and modeling elements, the other (Öst, 1996) addressed the question whether group size influences therapy outcome. Götestam (2002) investigated three kinds of OSTs in groups of spider phobic individuals where exposure was either delivered directly, indirectly or via a film clip. He reported modeled exposure, where only one participant and the therapist work together in front of the group or filmed modeling being both effective fear reducing strategies. However, direct exposure where each participant worked with his/her own spider proved slightly superior. However, Öst et al. (1997b) found a significant difference between the treatment effects of direct and indirect OST-group approaches with direct exposure yielding larger and more stable outcomes over time. Moreover, Öst (1996) showed that the effects of smaller groups (*n* = 3–4) in most measures did not differ significantly from those of larger groups (*n* = 7–8). However, there was a trend for the small group condition to result in better effects. In sum, small group OSTs proved feasible and effective, especially when employing direct exposure elements.

Furthermore, there was a non-clinical aspect encouraging us to invent an exposure based large-group treatment-protocol. Exposure treatments are often considered as an analog to extinction in laboratory fear studies (Hermans et al., 2006) and the construction of clinical exposure treatments has greatly benefitted from the results of experimental basic research on fear (Craske and Mystkowski, 2006). However, clinical trials are intensive with respect to recruitment-, cost-, and time-related issues; a reason for the gap between basic and applied clinical research. Therefore, feasible large-group treatment formats could enable direct and efficient transfer of mechanistic findings into clinical treatment research designs. Consequently, it would be easier to investigate the external validity of mechanisms identified under laboratory conditions.

Based on these insights, we developed an OST-protocol for a large group setting (LG-OST) that should contain well-evaluated OST-exposure strategies for specific phobias suitable for more than 50 participants. Following the concept of evidence-based psychotherapy (Norcross et al., 2006), in this Phase I open trial we primarily focused on feasibility aspects of LG-OST in a non-clinical sample of highly spider fearful individuals. Hence, this study does not contain a direct control group condition. However, with future research on LG-OST in mind, we compared the effects of a non-randomized LG-OST condition to a non-randomized single person OST (SP-OST). According to the recommendations of Öst (1989), SP-OST combines modeled and direct exposure elements and is routinely conducted in the same treatment center and accessible for the same cohort as LG-OST. We did not expect LG-OST to equal the recent ‘OST-gold standard.’ However, we aimed at bringing forth a first classification of LG-OST-results by comparing it to a one-session-single-person treatment condition (SP-OST). Furthermore we wanted to explore whether there are predictors for LG-OST success.

To the best of our knowledge, the delivery of a psychological treatment simultaneously to 50 or more individuals has never been conducted to date. Consequently, we were cautious of participants’ fear responses in a direct exposure setting with many spiders and their possible difficulties in dealing with them. Therefore, for the better control of fear responses, we included both live and filmed modeling modules in our LG-OST-protocol, although filmed modeling has been proven slightly less effective than direct exposure strategies.

## Materials and Methods

### Participants

For this Phase I study, initially 100 individuals registered for LG-OST online on a website established for this project. They were recruited by advertisements on local radio, newspapers and social networks. There were only two requirements to participate in the project: high subjective spider fear or avoidance and being full of age (18 years). Finally on the day of the group treatment, *N* = 78 participants, all Caucasian (67 female, 9 male) with a mean age of 34.65 (*SD* = 13.39) years showed up at the treatment-day where they gave their informed consent to attend LG-OST.

After the LG-OST, online-recruitment took place for SP-OST. Preconditions for participation in the SP-OST were the same as for LG-OST. Initially 35 individuals declared interest in SP-OST-participation. However, an appointment could be arranged only with 30 participants (27 female, 3 male) who were also all Caucasian, with a mean age of 27.40 (*SD* = 6.80) years.

Exclusion criteria for both LG-OST and SP-OST were allergic reactions to spider or insect bites or stings. Prior participants of the LG-OST were excluded from the SP-OST. The local Ethics Committee of the psychology faculty of the Ruhr-University Bochum, where the study was conducted, approved the study. Informed consent procedure was carried out with participants.

### Procedure

**FIGURE 1:**
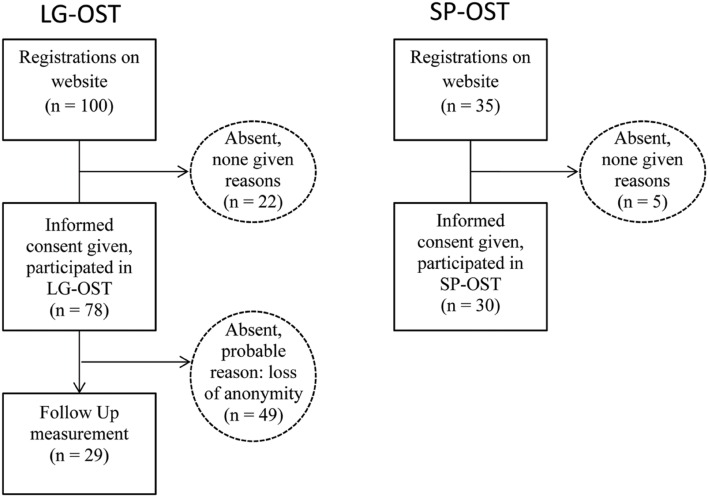
**Flow diagram of patients entering the study**.

Prospects were informed on our website that they would remain anonymous during the treatment. Prior to the intervention, participants were given an ID-number for data collection and assembling. Four postgraduate clinical psychologists, who were all well trained in spider phobia treatment, performed the LG-OST and SP-OST. Both conditions were preceded and followed instantaneously by a behavioral approach test (BAT). During the BAT, participants had to approach a living spider. Moreover, participants completed a set of questionnaires, assessing subjective and cognitive components of spider fear pre- and post-intervention. Before leaving, all participants were asked to voluntarily sign up and to be available for FU-measures. Twenty-nine participants (37.2%) registered (see **Figure 1**). After 8 months, we invited them for FU-measurement consisting of the same BAT and questionnaires.

### Treatment

#### LG-OST (Duration 120 Min)

Large group one-session exposure treatment was delivered to the patients in an auditorium of the Ruhr-University Bochum, consisting of three phases.

##### Psychoeducation phase (about 30 min)

After entering the auditorium, participants received information about spiders via a 20-min movie-clip. In this clip, a biologist specialized in research on spiders, targeted often-feared myths about spiders’ behavior, i.e., their potential aggressiveness, deceitfulness or toxicity.

Afterward, the psychotherapist explained the nature and utility of fear and its cognitive, behavioral and subjective consequences. He outlined the aim of the following treatment as a strategy to habituate to fear reactions and to reduce fear of spider’s behavior by learning to predict and control it. Participants were instructed that fear responses during the session would be normal and even useful because they could accelerate habituation effects.

##### Filmed modeling phase (about 50 min)

Exposure was delivered via video presentation introducing four spiders in sequence: a cellar spider *(Pholcus phalangioides)*, a garden spider *(Araneus diadematus)*, a long-legged male and a short-legged female barn funnel weaver *(Tegenaria atrica)*. The instructor asked participants to mentally describe all physical aspects of the spiders as precisely as possible as if they were to describe them to a blind person, thereby avoiding the use of negative predicates in their descriptions. Afterward, a direct modeled exposure, based on the recommendations of Öst (1989) was presented in the clip.

##### Live modeling phase (about 40 min)

After the video presentation, the psychotherapist asked one volunteer from the auditorium to repeat the program depicted in the video with a living barn funnel weaver. Other participants observed the process.

#### SP-OST (Duration 120 Min)

We presented the same movie clip with information about spiders as in the LG-OST-condition and participants received the same psycho-educative information as in LG-OST. All participants were treated based on the one-session-treatment concept of Öst (1989), which combines gradual exposure and modeling. According to their subjective fear reduction, participants indicated their willingness to proceed to the next step. The program was finished after the participant had reached the last step or after a total duration of 2 h.

### Measures

#### Behavioral Approach Test

A BAT was performed using a test with a living spider (*Tegenaria domestica).* At pre-, post-, and FU-measuring points, participants were instructed to undergo five steps: (1) To go through a 5 m long corridor and approach a desk with a well visible living barn funnel weaver captured in a drinking glass; (2) To touch the glass with the spider; (3) To place their other hand on the glass; (4) To lift the glass to their face; (5) To turn the glass upside down and touch the spider. It was scored whether a participant managed to cope with a respective step for at least 5 s.

#### Subjective Spider Fear Measures

The German version of the *Spider Phobia Questionnaire* (SPQ, Rinck et al., 2002) consists of 43 dichotomous items (yes/no), covering three dimensions of spider fear: vigilance (6 items), preoccupation (17 items) and avoidance-coping (10 items). Furthermore, the SPQ contains five items dealing with facts about spiders and five cognitive-behavioral items not being assigned to any scale. The authors reported a total mean score of *M* = 16.7 (±6.2) in highly spider fearful individuals. In our sample, internal consistency of the SPQ was good (Cronbachs’ α = 0.85) with the subscales ranging from α = 0.62 (vigilance) to α = 0.79 (preoccupation).

The German version of the *Fear of Spider Questionnaire* (FSQ, Rinck et al., 2002) consists of 18 items with 7-point Likert-scales (0 = not at all true; 6 = exactly true). The mean score of highly spider fearful persons was reported to be 58.7 (±22.2). Internal consistency (Cronbachs’ α = 0.96) and retest reliability (*r*_tt_ = 0.95) were reported as excellent. In our sample, internal consistency was Cronbachs’ α = 0.93.

The German *Spider Fear Screening* (Rinck et al., 2002) is a short self-rating instrument consisting of four items all conceiving the relevant DSM-IV criteria for spider phobia on a 7-point Likert-scale. The authors report mean scores of 1.6 (±1.2) for non-fearful individuals and 21.4 (±2.3) for individuals with a diagnosed spider phobia. It has a good internal consistency, Cronbach’s α = 0.92, and a retest reliability of *r*_tt_ = 0.88. We found a good reliability of Cronbach’s α = 0.82 of this four-item measure in our sample.

#### Dysfunctional Spider-Related Cognitions

The German version of the *Spider Belief Questionnaire* (Pössel and Hautzinger, 2003) consists of 48 items, measuring dysfunctional beliefs. The questionnaire consists of two groups of items measuring beliefs regarding (a) spider-related endangerment (five subscales: ‘Hunter and Victim’ i.e., spiders pursue the individual; ‘Unpredictability’; ‘Aggression’; ‘Multiplication’ [i.e., spider will grow and new spiders will show up]; ‘Territory’ [i.e., spiders will intrude the private sphere]) and (b) self-related reactions and coping (three subscales: Panic and uncontrollable reactions, Paralysis, Nightmares). Participants indicate the degree to which the respective statement applies to them (0–100). The authors of the German SBQ report an excellent internal reliability of Cronbach’s α = 0.98 with the subscales ranging from 0.82 to 0.96. Here, we found an internal consistency of Cronbach’s α = 0.95 for the total scale.

#### Clinical State

A German 21-item version of the *Depression Anxiety Stress Scale* (Lovibond and Lovibond, 1995) was used. This self-rating questionnaire measuring negative emotional status is answered on a 7-item Likert-scale. Besides the mean score, a set of seven items constitutes three separate scales: depression, anxiety and stress. Here, we found an internal consistency of Cronbach’s α = 0.90 for the total scale, with subscales ranging from Cronbach’s α = 0.90 (depression) to 0.78 (stress).

The German version of the *State-Trait Anxiety Inventory* (STAI, Laux et al., 1981) consists of two subscales, each describing emotional state in 20 statements at present (state) and during the last 2 weeks (trait version). Scores range from 20 (no anxiety) to 80 (high anxiety). Whereas the state-scale is highly sensitive for change, the trait-scale has a high retest reliability (*r*_tt_ < 0.96).

#### Global-Success-Rating (GSR)

On a 7-item Likert-scale, participants rate subjective state changes from 1 (much worse) to 7 (much better). A score of 4 indicates no subjective change.

### Statistical Analysis

Due to absence of premature attrition, analysis on change was carried out as completer analyses using repeated measures ANOVA. Between measures, *n* could slightly differ, i.e., due to falsely completed questionnaires. We calculated within-group effect sizes using Cohen’s *d* formula based on pooled standard deviations (Cohen, 1988). Moreover, partial eta-square values (η^2^) as measures for effect-size were given, for within- and between-group comparisons. In case of significant pre-treatment differences between LG-OST- and SP-OST- participants, repeated measures ANCOVA were used for between-group comparisons.

Only 37% of the participants returned for FU-assessment in the LG-OST-condition. To test weather drop-out was selective; we initially compared FU-completers and non-completers with univariate ANOVA for every pre-treatment and all outcome measures. Afterward, the stability of LG-OST-effects was analyzed with repeated measures ANOVA within FU-completers.

To reduce LG-OST outcome measures, we initially conducted a factor analysis choosing a non-rotated factor solution, including all pre-post change scores. Afterward, Pearson correlation-analyses were conducted to identify variables that were significantly associated with LG-OST-treatment outcome. Afterward, these variables were included in the multiple regression analysis. All analyses were conducted using the IBM SPSS Statistics program.

## Results

### Is LG-OST Effective in Reducing Spider Fear?

Regarding total scores, ANOVA showed highly significant decreases from pre- to post-treatment in all subjective spider fear measures and the BAT. With the exception of two subscales of the SPQ (Vigilance and Preoccupation), all subscale-changes were highly significant as well. For the total scores, the effect sizes (Cohen’s *d*) ranged from large effects *d* = 1.21 (SBQ) to small effects of Cohen’s *d* = 0.25 (SPQ), as depicted in **Table 1**.

**Table 1:** Means, SDs and effect strengths (Cohen’s *d*) of pre- to post changes of spider fear measures for LG-OST-condition.

	LG-OST (*n* = 78)			
	
	Pre	Post	Pre > Post	
			
	*M (SD)*	*M (SD)*	Statistics	*ES^#^* [95 % CI]
**Sample characteristics and clinical data**				
Age (years)	34.65 (13.39)	–	*–*	–
STAI-S	42.01 (5.08)	40.90 (5.52)	*F*(1,70) = 3.38^∗^, η^2^ = 0.05	0.21 [0.54 – (-0.12)]
STAI-T	41.52 (11.12)	–	*–*	–
DASS	16.36 (10.11)	–	*–*	–
**Spider fear measures**				
SBQ (total)	50.56 (21.00)	27.07 (17.24)	*F*(1,70) = 208,11^∗∗∗^, η^2^ = 0.75	1.21 [1.55 -0.83]
*SBQ subscales*				
Panic	66.81 (27.60)	37.07 (26.88)	*F*(1,70) = 153,15^∗∗∗^, η^2^ = 0.69	1.09 [1.44 -0.74]
Paralyze	34.52 (35.21)	20.68 (29.50)	*F*(1,70) = 24.55^∗∗∗^, η^2^ = 0.26	0.43 [0.76 -0.09]
Nightmare	57.87 (30.17)	31.03 (28.17)	*F*(1,70) = 85.00^∗∗∗^, η^2^ = 0.55	0.92 [1.27 -0.57]
Unpredict.	73.41 (21.07)	47.82 (22.20)	*F*(1,70) = 138,05^∗∗∗^, η^2^ = 0.66	1.18 [1.54 -0.83]
Territory	46.28 (25.25)	22.79 (20.79)	*F*(1,70) = 85.68^∗∗∗^, η^2^ = 0.55	1.02 [1.37 -0.67]
Victim.	28.65 (24.35)	7.69 (11.79)	*F*(1,70) = 82.06^∗∗∗^, η^2^ = 0.54	1.01 [1.45 -0.74
Multipl.	23.41 (28.22)	11.17 (16.29)	*F*(1,70) = 25.78^∗∗∗^, η^2^ = 0.27	0.53 [0.87 -0.20]
Aggress.	22.90 (28.92)	6.54 (11.65)	*F*(1,70) = 38.60^∗∗∗^, η^2^ = 0.36	0.74 [1.08 -0.40]
SFS (total)	19.51 (5.22)	15.43 (6.42)	*F*(1,73) = 77.86^∗∗∗^, η^2^ = 0.52	0.69 [1.01 -0.36]
FSQ (total)	61.83 (25.24)	40.01 (23.41)	*F*(1,70) = 96,35^∗∗∗^, *η*^2^ = 0.58	0.88 [1.22 -0.53]
SPQ (total)	17.73 (6.11)	16.00 (7.30)	*F*(1,72) = 14.85^∗∗∗^, η^2^ = 0.17	0.25 [0.57 – (-0.08)]
*SPQ subscales*				
Vigilance	4.26 (4.14)	4.13 (1.49)	*F*(1,72) = 1.11, η^2^ = 0.02	–
Preoccupation	6.60 (3.61)	6.13 (4.28)	*F*(1,71) = 3.56, η^2^ = 0.05	–
Avoid.Cop	6.88 (2.39)	5.90 (2.72)	*F*(1,71) = 24.41^∗∗∗^, *η*^2^ = 0.26	0.38 [0.71 -0.01]
BAT steps	1.04 (1.41)	2.68 (1.37)	*F*(1,71) = 105.12^∗∗∗^, *η*^2^ = 0.60	-1.18 [(-1.53) – (-0.83)]
GSR	–	5.13 (0.94)	–	–


*STAI, State-Trait Anxiety Inventory; DASS-21, Depression Anxiety Stress Scale; SBQ, Spider Belief Questionnaire; Unpred., Unpredictability; Multipl., Multiplication; Aggress., Aggression; SFS, Spider Fear Screening; FSQ, Fear of Spider Questionnaire; SPQ, Spider Phobia Questionnaire; GSR, Global Success Rating; Preoccupat., Preoccupation; Avoid.Cop, Avoidance-Coping; BAT, Behavioral Approach Test; ^∗^*p* < 0.05, ^∗∗^*p* < 0.01, ^∗∗∗^*p* < 0.001; ^#^Effect sizes not reported when pre- to post changes were not significant.*

### What Are Predictors for LG-OST-Outcome?

To reduce the dimensions of therapy outcome, we initially conducted a factor-analysis with non-rotated factors including all pre to post differences concerning subjective spider fear- (SFS, SBQ, SPQ, FSQ), behavioral spider fear- (BAT) and state-fear (STAI-State) measures. A two factor- solution (Eigenwerte > 1) explained 66.09% of the total variance. All spider fear measures loaded high (all loads > 0.67) on the first factor ‘spider fear decrease’ which explained 48.40% of the total variance. The STAI-State loaded high (0.92) on the second factor ‘state-fear decrease,’ explaining 17.69% of the total variance. No spider fear measure loaded higher than 0.30 (BAT) on the second factor.

Subsequently, we conducted correlation analyses between ‘spider fear decrease’ factor-scores and age, clinical baseline-status (DASS, STAI-Trait) and all spider fear pre scores in the LG-OST condition. A significant result was shown for age (*r* = -0.28, *p* < 0.05) with younger participants benefitting more than older ones. More dysfunctional spider-related cognitions at baseline in the SBQ (*r* = 0.27, *p* < 0.05) and higher subjective spider fear levels in FSQ (*r* = 0.37, *p* < 0.05) were both positively associated with treatment outcome. However, there were no significant correlations between ‘spider fear decrease’ and clinical status (DASS, STAI-T).

A multiple regression analysis with stepwise inclusion of FSQ, age and SBQ yielded best variance explanation (*R*^2^ = 0.21) for a model combining FSQ (β = 0.36, *t* = 3.23, *p* < 0.01) and age (β = -0.26, *t* = -2.34, *p* < 0.05).

### Are the Effects of LG-OST Stable Over Time?

Twenty-nine LG-OST-participants (85.7% female, 14.3% male) returned for FU-measures. Mean time between post- and FU-measure was 8.96 months (*SD* = 1.79). To investigate selective drop-out effects, we compared gender with χ^2^-test and all baseline measures for FU-completers and non-completers (*n* = 49) with univariate ANOVA. With the exception of one subscale (Aggression) of the SBQ, where FU-completers reported significantly more dysfunctional beliefs [*F*(1,75) = 4.33, *p* = 0.041], there were no significant differences between groups at baseline-assessment neither in regard to sociodemographics and clinical state, nor spider-fear measures. The same non-significant results were found for all pre- to post-change scores. FU-completers and non-completers did not differ in any of them (all *p* > 0.05). Hence, we assume that the post-treatment to FU drop-out was random within the LG-OST-condition. As depicted in **Table 2**, with the exception of SPQ, FU-completer analyses showed no significant changes from post-treatment to FU, indicating time-stable effects of LG-OST.

**Table 2:** Means, SDs, CI’s and effect strengths (Cohen’s *d*) of post- to FU changes of spider-fear measures for LG-OST-participants completing FU-measures.

	LG-OST (*n* = 29)				
			
	Pre	Post	FU		
	*M (SD)*	*M (SD)*	*M (SD)*	Statistics	*ES^#^* [95% CI]
SBQ total	50.63 (18.57)	28.90 (18.07)	31.54 (19.97)	*F*(1,27) = 0.67, n.s.	*–*
*SBQ subscales*					
Panic	68.70 (25.70)	41.19 (29.73)	44.14 (32.72)	*F*(1,27) = 0.57, n.s.	*–*
Paralyze	37.52 (35.95)	24.92 (33.96)	18.29 (25.24)	*F*(1,27) = 2.18, n.s.	*–*
Nightmare	61.79 (27.70)	39.70 (31.47)	43.87 (34.58)	*F*(1,27) = 0.53, n.s.	*—*
Unpred.	77.21 (14.39)	49.66 (20.71)	56.37 (24.70)	*F*(1,27) = 2.07, n.s.	*–*
Territory	44.62 (24.93)	23.63 (21.74)	29.22 (25.35)	*F*(1,27) = 1.24, n.s.	*–*
Victim.	25.32 (25.75)	6.43 (8.82)	8.13 (9.20)	*F*(1,27) = 0.83, n.s.	*–*
Multipl.	21.54 (29.93)	7.99 (15.62)	7.90 (13.32)	*F*(1,27) = 0.97, n.s.	*–*
Aggress.	14.02 (21.49)	3.84 (8.84)	3.79 (7.80)	*F*(1,27) = 0.00, n.s.	*–*
SFS	20.86 (3.57)	16.66 (5.68)	15.24 (6.47)	*F*(1,28) = 1.65, n.s.	*–*
FSQ	62.82 (24.71)	42.89 (24.79)	38.25 (27.25)	*F*(1,27) = 1.04	*–*
SPQ total	18.35 (6.22)	17.00 (7.08)	13.97 (6.26)	*F*(1,28) = 11.78^∗∗^, η^2^ = 0.30	0.45 [0.98 -0.07]
*SPQ subscales*					
Vigilance	4.28 (1.41)	4.21 (1.42)	4.03 (1.72)	*F*(1,28) = 0.75, n.s.	–
Preoccupat.	7.21 (3.75)	6.79 (4.02)	5.07 (3.47)	*F*(1,28) = 9.29^∗∗^, η^2^ = 0.25	0.46 [0.98 -0.06]
Avoid.Cop.	6.86 (2.45)	6.00 (2.96)	4.86 (2.37)	*F*(1,28) = 6.43^∗^, η^2^ = 0.19	0.43 [0.95 -0.10]
BAT steps	1.15 (1.49)	2.73 (1.37)	2.58 (0.61)	*F*(1,25) = 0.49	*–*
GSR	–	5.02 (1.13)	5.36 (0.88)	*F*(1,25) = 1.44	*–*


*SBQ, Spider Belief Questionnaire; Unpred., Unpredictability; Multipl., Multiplication; Aggress., Aggression; SFS, Spider Fear Screening; FSQ, Fear of Spider Questionnaire; SPQ, Spider Phobia Questionnaire; Preoccupat., Preoccupation; Avoid.Coping, Avoidance-Coping; BAT, Behavioral Approach Test; GSR, Global Success Rating, ^∗^*p* < 0.05, ^∗∗^*p* < 0.01, ^∗∗∗^*p* < 0.001; ^#^Effect sizes not reported when post- to FU-changes were not significant.*

The pre to post change-effects within the SPQ were small (see **Table 1**). However, SPQ was the only measure where LG-OST-participants showed a significant decrease from post-treatment to FU.

### Are the Post-treatment Effects of LG-OST within a Non-randomized Sample Equal to those of a Non-randomized One-Session Single Training (SP-OST)?

To offer a clearer classification of LG-OST-outcomes, we compared LG-OST effects with those of SP-OST, where 30 individuals were treated in a one session-session single treatment. For SP-OST sample characteristics, clinical state and spider fear see **Table 3**.

**Table 3:** Means, SDs and effect strengths of pre- to post changes (Cohen’s *d*) of spider fear measures for SP-OST.

	SP-OST (*n* = 30)			
	
	Pre	Post	Pre > Post	
			
	*M (SD)*	*M (SD)*	Statistics	*ES^#^* [95% CI]
**Sample characteristics and clinical data**				
Age (years)	27.39 (6.80)	–	–	–
STAI-S	41.87 (4.13)	40.97 (4.66)	*F*(1,29) = 0.33, n.s.	–
STAI-T	39.50 (8.24)	–	–	
DASS	11.80 (6.92)	–	–	–
SBQ (total)	49.41 (18.95)	23.24 (14.79)	*F*(1,29) = 78.89^∗∗∗^, η^2^ = 0.73	1.54 [2.11 -0.96]
*SBQ subscales:*				
Panic	66.98 (29.23)	31.13 (27.48)	*F*(1,29) = 56.76^∗^*^∗∗^*, η^2^ = 0.66	1.26 [1.82 -0.71]
Paralyze	31.82 (32.49)	15.89 (21.66)	*F*(1,29 = 14.39*^∗∗∗^*, η^2^ = 0.33	0.58 [1.09 -0.06]
Nightmare	59.60 (30.27)	30.00 (27.48)	*F*(1,29) = 41.67*^∗∗∗^*, η^2^ = 0.59	1.02 [1.56 -0.49]
Unpred.	67.32 (19.88)	39.28 (17.03)	*F*(1,29) = 83.53^∗^*^∗∗^*,η^2^ = 0.74	1.52 [2.09 -0.94]
Territory	52.21 (28.68)	19.52 (18.18)	*F*(1,29) = 43.89*^∗∗∗^*, η^2^ = 0.60	1.34 [1.90 -0.78]
Victim.	22.84 (23.00)	3.04 (6.19)	*F*(1,29) = 24.69*^∗∗∗^*, η^2^ = 0.46	1.18 [1.72 -0.06]
Multipl.	20.78 (23.15)	5.94 (10.75)	*F*(1,29) = 22.36^∗∗∗^, η^2^ = 0.44	0.82 [1.35 -0.30]
Aggress.	21.97 (23.23)	4.23 (8.20)	*F*(1,29) = 23.27*^∗∗∗^*,η^2^ = 0.45	1.02 [1.56 -0.48]
SFS (total)	20.11 (4.08)	12.04 (5.59)	*F*(1,29) = 83.93^∗∗∗^, η^2^ = 0.74	1.65 [2.24 -1.06]
FSQ (total)	66.71 (23.85)	34.68 (19.34)	*F*(1,29) = 95.50^∗∗∗^, η^2^ = 0.77	1.48 [2.05 -0.90]
SPQ (total)	18.77 (6.36)	16.20 (6.86)	*F*(1,29) = 9.34^∗∗^, η^2^ = 0.24	0.38 [0.89 -0.13]
*SPQ subscales:*				
Vigilance	4.00 (1.60)	4.20 (1.37)	*F*(1,29) = 1.53, η^2^ = 0.05	–
Preoccup.	7.47 (3.94)	6.40 (4.68)	*F*(1,29) = 5.69^∗^, η^2^ = 0.16	0.25 [0.76 – (-0.26)]
Avoid.Coping	7.30 (2.22)	5.60 (3.73)	*F*(1,29) = 7.43^∗^, η^2^ = 0.20	0.55 [1.07 -0.04]
BAT steps	0.23 (0.61)	3.32 (0.76)	*F*(1,29) = 447.10^∗∗∗^, *η^2^* = 0.94	-4.48 [(-3.54) – (-5.43)]
GSR	–	5.97 (0.78)	–	*–*


*STAI, State-Trait Anxiety Inventory; DASS, Depression Anxiety Stress Scale; SBQ, Spider Belief Questionnaire; Unpred., Unpredictability; Multipl., Multiplication; Aggress., Aggression; SFS, Spider Fear Screening; FSQ, Fear of Spider Questionnaire; SPQ, Spider Phobia Questionnaire; Preoccupat., Preoccupation; Avoid.Cop, Avoidance-Coping; BAT, Behavioral Approach Test; ^∗^*p* < 0.05, ^∗∗^*p* < 0.01, ^∗∗∗^*p* < 0.001; GSR, Global Success Rating; ^#^Effect sizes not reported when pre- to post-changes were not significant.*

Gender-distributions did not differ between the LG-OST- and SP-OST-conditions (χ^2^ = 0.07, *p* = n.s.). However, participants of the LG-OST-condition were significantly older than SP-OST-participants [*F*(1,105) = 7.49, *p* < 0.01] and expressed higher mental distress in the German DASS-21 [*F*(1,105) = 5.12, *p* < 0.05].

To this end, we used ANCOVA partialing-out the age and DASS-differences for all spider fear comparisons between groups. Initially, we analyzed whether there were baseline differences between groups in regard to spider fear. However, for none of the subjective measures differences were found (all age- and mental distress-adjusted means *p* > 0.05). BAT at pre-treatment showed that participants of the LG-OST-group approached the spider closer than SP-OST-participants [*F*(1,99) = 10.27, *p* < 0.01, η^2^ = 0.09).

**FIGURE 2:**
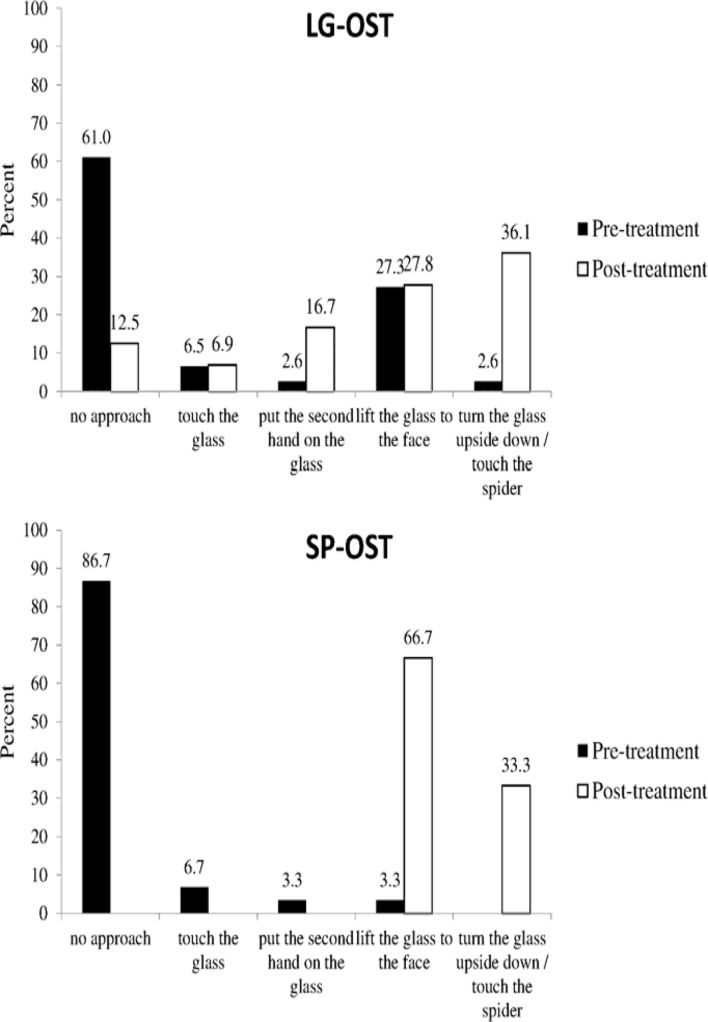
**Pre- and post-treatment approach in the BAT for the LG-OST and SP-OST condition**.

Regarding differential pre- to post changes (group × time interactions), outcomes differed between groups in the FSQ [*F*(1,94) = 6.70, *p* < 0.05, η^2^ = 0.07] and in SFS [*F*(1,97) = 16.70, *p* < 0.001, η^2^ = 0.15], with SP-OST resulting in larger effects than LG-OST in both measures. In the SPQ [*F*(1,95) = 0.91, *p* = n.s.] and in SBQ [*F*(1,96) = 0.42, *p* = n.s], however, changes did not significantly differ between groups. The two groups differed highly significantly in approaching the spider after treatment [*F*(1,94) = 15.98, *p* < 0.001], with a better performance in SP-OST than LG-OST-participants (see **Figure 2**).

In regard to subjective treatment success (GSR – Global Success Rating), SP-OST participants expressed higher global treatment success [*F*(1,99) = 18.53, *p* < 0.001] with SP-OST-ratings being superior.

## Discussion

In this Phase I study we investigated whether a one session treatment, based on an indirect exposure approach is feasible and efficacious in a large group setting. Preliminary results were promising: in terms of mere feasibility, our concerns regarding patients’ doubts about effectiveness of the treatment and a possibly consequent weak participant-inflow as well as considerations regarding a potential mass panic or aggravation during the session all proved ungrounded.

We observed a reduction of spider fear symptoms on subjective and behavioral levels with large effect sizes regarding dysfunctional beliefs, i.e., the SBQ and behavioral symptoms (BAT) and medium effect sizes in measures focusing on subjective fear, i.e., the FSQ and SFS. Moreover, the completer analysis indicated the stability of LG-OST-effects within the 8-month FU- interval. In the SPQ, however, there were only small post-treatment effects with a point change of 1.7 or 9.6%. Similar to our LG-OST, Öst et al. (1997b) investigated the effects of direct observation of a modeled exposure and indirect observation of modeled exposure via a movie-clip in two different conditions in a small group setting. They also applied the SPQ as an outcome measure and reported a change of 19.7% from pre- to post-treatment for the direct observation and 24.8% for the indirect observation condition. However, the participants of the Öst et al. (1997b) study were spider phobic individuals reporting 30.2% higher spider fear at baseline assessment in the direct observation condition (Mean score = 25.4) and 16.0% higher spider fear in the indirect observation condition (Mean score = 21.1) than LG-OST-participants (Mean = 17.7). Although the reported post-treatment effects were larger, SPQ-fear levels of one condition were higher (20.4 for direct observation) or only slightly lower (15.1 for indirect observation) than the levels reported in LG-OST (16.00) after treatment. Moreover, patients of the Öst et al. (1997b) study were younger (29.50 vs. 34.65 years) and reported higher baseline fear levels than LG-OST-participants. Younger age and higher baseline fear both proved as factors that foster treatment outcome in LG-OST. Furthermore, Öst et al. (1997b) collected post-treatment data 1 week after treatment, whereas LG-OST-participants completed the SPQ directly after the intervention. Many items of the SPQ inquire behavioral and subjective spider fear symptoms in specific situations, i.e., ‘If you find a spider in the bath, would you, say, use a shower to wash the spider down the plughole?’ Therefore, it is probable that LG-OST-effects were not directly depicted in the post assessment of the SPQ, because participants could not have tested or experienced possible symptom changes by then. SPQ being the only measure to decrease significantly from post-treatment to FU in LG-OST underlines this point: with a total decrease of 23.9% from pre-treatment to FU-assessment in the LG-OST-condition, SPQ outcomes of LG-OST equaled those of the small-group condition receiving direct modeled exposure observation (24.0% from pre to FU) reported by Öst et al. (1997b).

In sum, LG-OST led to reductions of subjective and BAT symptoms in highly spider fearful individuals comparable to effects reported of interventions using indirect exposure strategies in smaller groups. The follow-up (FU) data suggest that the reductions in spider fear following from treatment are sustained in the long-term. However, in a group (Öst, 1996; Öst et al., 1997b; Götestam, 2002) as well as in single-settings (Hellström and Öst, 1995) strategies combining therapist-directed direct and modeled exposure were more efficient than indirect- or self-exposure. Accordingly, also in our SP-OST-condition, participants reported larger pre- to post-treatment effects than in LG-OST in half of the applied subjective fear measures and especially in the BAT.

The study exhibits several limitations: We did not include an untreated control group in this feasibility-trial, thereby considering the danger of confounding post-treatment effects with effects of repeated measurement or a regression toward the mean in LG-OST. Furthermore, because randomization was not used, direct comparisons between the conditions are questionable. These facts clearly restrict the results on comparable effectiveness. Furthermore it is remarkable, that only 29 out of 78 participants (37%) participated in the FU assessment. As mentioned, we strived to minimize possible doubts to participate in large group training by guarantying anonymity. However, participants needed to actively waive anonymity to participate in FU-measures. Nevertheless, we proved that drop-out was unlikely to be systematic and, therefore, the reported FU-effects can be considered as suitable indicators of long-term effects of LG-OST.

However, our positive outcomes regarding feasibility of indirect exposure strategies in LG-OST in highly fearful individuals should promote research on large group exposure treatments. As mentioned, symptom-severity resulting from different specific phobias is highly variable and future research should highlight the question whether large-group settings may also be a treatment option in a sample of diagnosed spider phobic individuals or individuals suffering from even more restrictive phobic disorders such as dental phobia or injection phobia. Future efforts targeting spider phobia should eliminate the limitations of the present study, i.e., by using living spiders and by comparing the large group effects to a no-treatment control group condition. Hence, future research should address the question whether the effects of the ‘gold-standard’ intervention also sustain in a large group setting. If so, considering the very high efficiency as well as cost-effectiveness, LG-OST would represent a very valuable treatment tool, e.g., as an intermediate step within a framework of stepped care of phobic fears. Our results suggest that a LG-OST protocol might sufficiently address the needs for fear treatment of many but not all of our participants. Thus, within a stepped care model, patients might first be referred to very low intensive treatments such as bibliotherapy or self-help books, those who still need additional treatment might then progress to LG-OST as the next, moderately intensive treatment option, while only LG-OST non-responders may finally progress to individual treatment formats.

Moreover, clinical usability and high efficiency of LG-OST could promote research. We still do not know whether the mechanisms identified as potentially underlying fear in lab-based research can be translated into a more natural fear-related context. Highly standardized treatment delivery, as warranted in LG-OST could be a feasible component of future mechanistic fear studies in a clinical context. The comparatively high variance in the LG-OST outcome measures might even suit basic research.

## Conclusion

A large group OST based on indirect, modeled exposure proved feasible in highly fearful participants. The effectiveness of the LG-OST-protocol equaled the effects of previously reported small group-treatments applying comparable delivery of exposure in individuals with spider phobia. The current ‘gold standard’ in the treatment of phobic fear consists of a combination of direct and modeled exposure and yields larger effects than LG-OST. However, if more direct exposure elements can be successfully integrated into large group treatments, due to their very high efficiency they may prove to be valuable in treating patients with phobia, e.g., within a stepped care approach.

## Author Contributions

Conceived and designed the study: AW, JM; DA, MK, and AM; Analyzed the data: AW; Wrote the paper: AW, TT, DA.

## Conflict of Interest Statement

The authors declare that the research was conducted in the absence of any commercial or financial relationships that could be construed as a potential conflict of interest.
